# Clinical application of retrograde sural neurofasciocutaneous flap repair combined with jingulian capsules to treat foot and ankle soft tissue defects

**DOI:** 10.12669/pjms.38.1.4546

**Published:** 2022

**Authors:** Zhiwei Hao, Shan Tian, Changqing Hu, Yan Jia

**Affiliations:** 1 Zhiwei Hao, The Second Department of General Surgery, Baoding First Central Hospital, Baoding, Hebei 071000, China; 2 Shan Tian, Medical Imaging Department, Baoding First Central Hospital, Baoding, Hebei 071000, China; 3 Changqing Hu, The Fifth Orthopaedics Department, Baoding First Central Hospital, Baoding, Hebei 071000, China; 4 Yan Jia, Physical Examination, Baoding First Central Hospital, Baoding, Hebei 071000, China

**Keywords:** Retrograde sural neurofasciocutaneous flap repair, Jingulian capsules, Soft tissue defect in the foot and ankle, Combination therapy

## Abstract

**Objectives:**

To observe and analyse the efficacy of retrograde sural neurofasciocutaneous flap repair combined with Jingulian capsules to treat foot and ankle soft tissue defects.

**Methods:**

One hundred and eighty patients with foot and ankle soft tissue defects were enrolled in the study from January 2016 to June 2019 in The Second Department of General Surgery,Baoding First Central Hospital. They were divided into a study group and a reference group with the same case number. The former group was provided combination treatment, i.e. retrograde sural neurofasciocutaneous flap repair combined with Jingulian capsules; the latter group was given vacuum sealing drainage. Then, the treatment outcomes of the two groups were compared.

**Results:**

The study group needed fewer dressing changes, less preoperative preparation time and antibiotic use than the reference group, *p*<0.05. The study group had a significantly lower incidence of wound infections and flap necrosis than the reference group, *p*<0.05. The study group was significantly superior to the reference group regarding ankle function scores and the pain visual analogue scores (VAS) *p*<0.05.

**Conclusions:**

Retrograde sural neurofasciocutaneous flap repair combined with Jingulian capsules is a protocol that improves efficacy for soft tissue defects in the foot and ankle, which are worthy of promotion and practice.

## INTRODUCTION

The prevalence of soft tissue defects has gradually increased due to increased health awareness and high energy exercise. Common ankle and foot soft tissue defects are often accompanied by bone and tendon exposure and become infected easily. So, their treatment is troublesome.^1^-^3^ The foot and ankle have thinner subcutaneous tissue than other regions and a relatively poor blood supply. Hence, foot and ankle trauma can easily lead to soft tissue defects while increasing the probability of deep tissue exposure. If not timely and effectively treated, they can lead to bone and tendon exposure and osteomyelitis, increasing the risk of disability.^4^,^5^ In recent years, surgery has been the preferred treatment modality for foot and ankle soft tissue defects. This study observed the efficacy of retrograde sural neurofasciocutaneous flap repair combined with Jingulian capsules to treat foot and ankle soft tissue defects.

## METHODS

**Fig.1:**
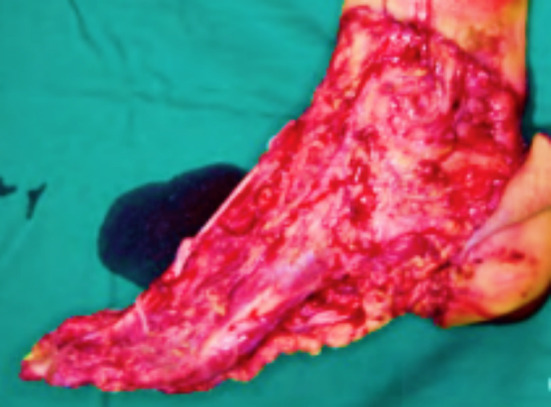
Preoperative patient.

One hundred and eighty patients with foot and ankle soft tissue defects were enrolled in the study (Fig.1) to receive treatment from January 2016 to June 2019, The study was performed at The Second Department of General Surgery,Baoding First Central Hospital. The patients were randomly divided into the study group and the reference group, with 90 patients in each. The average age was 46.25 ± 1.22 years. The study group had 46 males and 44 females, with an average age of 45.13 ± 1.70 years. The study group had 48 males and 42 females, with an average age of 46.18 ± 1.20. The causes of injury were 142 cases of traffic accident injuries and 38 cases of contusion injuries. The defect area was 6 cm × 5 cm - 12 cm × 8 cm. The data of the two groups were comparable, *p*>0.05.

### Ethical Approval:

The study was approved by the Institutional Ethics Committee of Baoding First Central Hospital (No. [2021]061; date: May 27^th^, 2021), and written informed consent was obtained from all participants

The study group received retrograde sural neurofasciocutaneous flap repair combined with Jingulian capsules. The retrograde sural neurofasciocutaneous flap repair operation allowed the patient to maintain a correct and comfortable prone position for combined spinal-epidural anaesthesia. Before the operation, Doppler was used to detect peroneal perforator, minimum septocutaneous perforator and lateral retromalleolar perforator. The line from the midpoint of the lateral malleolus tip and posterior margin of the tendon to the midpoint of popliteal space is the flap’s axis. The rotation point was about 0.5–3cm above the lateral malleolus. The distance from the rotation point to the farthest end of the defect was measured. The flap was drawn on the posterolateral side of the crus, which was ten per cent larger than the actual wound surface. The affected limb was lifted by 40° without exsanguinations, and a pneumatic tourniquet was set on the upper thigh. After conventional disinfection of the surgical drape, a cut was made on the proximal side of the posterior lateral flap of the crus until the deep fascia. The sural nerve and the small saphenous vein were cut. Next, respective ligation on both ends of the small saphenous veins was performed. Slowly spread to the distal end of the lateral malleolus tip along the nerve vessels. Separate the peroneal perforator, minimum septocutaneous perforator and lateral retromalleolar perforator, lateral vessel perforator with the protection given. The pedicle of the rotation flap was as narrow as 2-cm. For a larger flap or distal affected area, the distal end of the flap was made into the subdermal vascular net flap. The pedicle’s small saphenous vein was cut, and the two ends were ligated. The sural nerve’s proximal end was anastomosed to the cutaneous nerve. The flap thickness was adjusted according to the soft tissue defect site.^6^ Meanwhile, the subjects were orally administered Jingulian capsules (produced by Guizhou Yibai Pharmaceutical Co., Ltd., national medicine permission number: Z20123051), two tablets each time, three times a day with warm water after meals. The treatment was continued for one month. Jingulian capsules are a potent Chinese medicine that is yellow-brown to reddish-brown powder. Jingulian has a slight flavour that is slightly bitter. It is made of ingredients such as radix gaultheriae, Schefflera kwangsiensis, sargentgloryvine stem, Alangium platanifolium and Psammosilene tunicoides. Jingulian can dispel wind and eliminate dampness, reduce swelling and ease pain. It is used for joint swelling and pain and inhibited bending and stretching caused by rheumatic arthralgia.

### Reference group Treatment:

The reference group was treated with vacuum sealing drainage. The necrotic tissue was removed, and the vacuum sealing drainage material was cut as appropriate to cover the wound area and applied to the wound surface. The surrounding normal skin was sutured and fixed, and the silicone drainage tube was fixed inside. In the case of deep inner dead space, an irrigation tube was placed in the device. The vacuum sealing drainage material plus the wound surface was sealed with the biological semi-permeable membrane. The silicone drainage tube was connected to the vacuum extractor for one week’s vacuum aspiration under control pressure at 120–250 mmHg. After one week, the dressing was removed to observe the degree of skin wound infection. If the infection degree was up to the standard, the second-stage flap repair could be implemented. Otherwise, the vacuum sealing drainage material was replaced for further vacuum drainage until the skin wound was fresh.^7^,^8^

### Observation Indicators:

The preoperative preparation time, dressing change time, antibiotic use and the length of hospital stay were compared between the two groups. At the same time, half a year’s follow-up was performed to count the two groups’ complication rates (wound infection, flap oedema, flap necrosis, others). Ankle function was assessed using Kofoed scoring criteria, with a maximum score of 100.^9^ A lower score meant more severe ankle dysfunction. The pain degree was evaluated by a visual analogue scale (VAS) with 0–10 points. A high VAS score indicated severe pain.

### Statistical Analysis:

Using SPSS 21.0 statistical software, the measurement data were expressed as mean ± average (x ¯±s), and the count data were expressed by (n, %). A t and X2 were used for comparisons between groups. A *p*<0.05 indicated statistical significance.

## RESULTS

**Fig.2:**
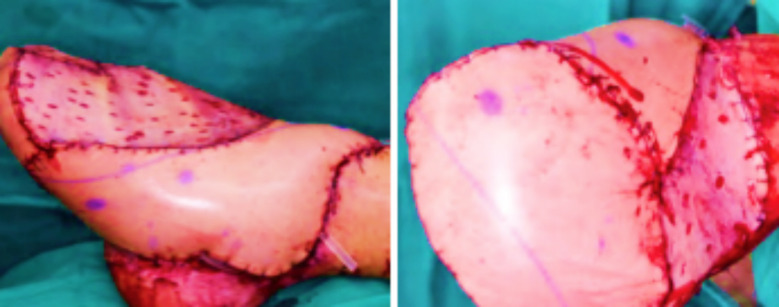
Immediate lateral and plantar appearance of a patient in the study group after repair.

**Fig.3:**
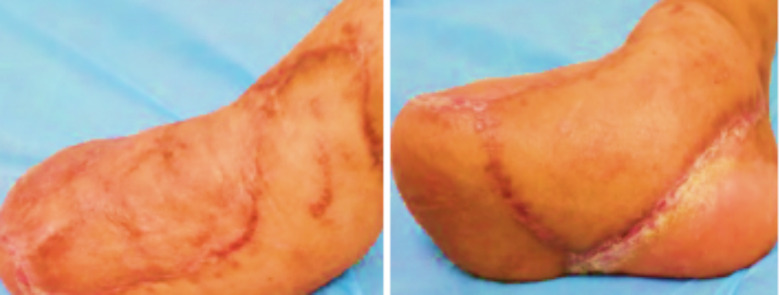
Lateral and plantar appearance of a patient in the study group at six months after the operation.

The study group’s treatment-related indicators were significantly better than the reference group, *p*<0.05 Table-I. Fig.2 and Fig.3 During the follow-up period, the study group’s ankle function and pain VAS scores were significantly better than the reference group, *p*<0.05 (Table-II).

**Table I:** Comparison of treatment-related indicators between the two groups.

Group	Preoperative preparation time (d)	Dressing change time (time)	Antibiotic use (d)	Length of hospital stay (d)	Complication rate (%)
Study Group	7.24 ± 2.10	3.55 ± 0.95	5.42 ± 1.20	16.59 ± 3.28	6(6.67)
Reference group	14.58 ± 2.83	8.69 ± 0.24	10.88 ± 1.29	28.95 ± 4.03	23(25.56)
X^2^	5.60	6.77	10.28	14.31	10.23
p	<0.05	<0.05	<0.05	<0.05	<0.05

**Table II:** Comparison of ankle function score and pain VAS score between the two groups.

Group	Case number	Ankle function score (points)	Pain VAS score (points)
Study group	90	90.28 ± 5.48	3.21 ± 0.38
Reference group	90	78.60 ± 3.27	6.24 ± 0.90
X^2^		18.93	6.55
p		<0.05	<0.05

## DISCUSSION

A sural neurofasciocutaneous flap is a flap that uses the sural nerve’s nutrient blood vessel as its nutrient blood vessel.^10^ There is a need to separate and then anastomose the blood vessels during the flap incision.^11^ The main crus blood vessels are not affected, so the nutrient blood vessels’ distribution is relatively stable. The correct application based on the strict selection of indications is superior to the free flap or island flap in repairing ankle and foot soft tissue defects.^12^,^13^ The sural nerve nutrient vascular pedicle flap causes no damage to the main blood vessels. No anastomosis of blood vessels is needed. So, the function will not be significantly affected. Meanwhile, it can restore the partial sensation of the ankle and the foot, which dramatically affects weight-bearing and foot sensation. The skin texture is similar to the affected skin and relatively wear-resistant.^14^,^15^ Moreover, the rotation point of the flap pedicle is moved down to expand the flap repair range. The flap’s sufficient blood supply enables flap thickness adjustment, as appropriate, for the soft tissue defect. The flap has a high survival rate and uncomplicated operation, which positions it as the primary choice for repairing ankle and foot soft tissue defects.^16^,^17^

Jingulian capsules are mainly composed of traditional Chinese medicines, such as radix gaultheriae, Schefflera kwangsiensis, sargentgloryvine stem, Alangium platanifolium and Psammosilene tunicoides, which play a role in relieving swelling and pain, dispelling wind and eliminating dampness. Radix gaultheriae has a sweet flavour and is pungent and warm. Acting on the lung, liver and kidney channel, it dispels wind and eliminates dampness, promotes blood circulation to remove the meridian obstruction. Modern research has found that the drug contains methyl salicylate whose oral administration has antipyretic, pain relief, anti-rheumatic effects.^18^ In addition, Schefflera kwangsiensis is neutral or cool with a bitter taste. It acts on the kidney, large and small intestine, and plays a role in expelling wind and dampness and relieving swelling. These actions can be beneficial for rheumatism, oedema and traumatic injury.^19^ Sargentgloryvine stem has a bitter taste and neutral nature. It acts on the large intestine and liver, can eliminate toxins and resolve the carbuncle, relax muscles and tendons, activate blood circulation, kill insects and dispel wind. Alangium platanifolium is spicy and slightly warm. It acts on the liver, kidneys, and heart. It can dispel wind and remove dampness, relax muscles and tendons, activate blood circulation, dispel stasis and alleviate pain. Psammosilene tunicoides is bitter, warm and toxic. It acts on the liver and can dispel the wind, alleviate pain, dispel stasis and stop bleeding.^20^

The study results showed that the study group needed fewer dressing changes, less preoperative preparation time and antibiotic use than the reference group, *p*<0.05. The study group had a significantly lower incidence of wound infections and flap necrosis than the reference group, *p*<0.05. The study group had significantly better ankle function scores and pain VAS scores (*p*<0.05) than the reference group. These results demonstrated that retrograde sural neurofasciocutaneous flap repair combined with Jingulian capsules effectively treat foot and ankle soft tissue defects.

### Limitations of this study

There are still some limitations in this study. First of all, the sample size of this study is not large enough. If the sample size can be further expanded, the conclusion may be more convincing. In addition, we only analyzed the cases included in our hospital, which may not be representative enough. We look forward to a multi-center study in the future to reach more comprehensive conclusions.

## CONCLUSIONS

Retrograde sural neurofasciocutaneous flap repair combined with Jingulian capsules is a protocol that improves treatment efficacy for foot and ankle soft tissue defects, which is worthy of promotion and practice. However, during the application of retrograde sural neurofasciocutaneous flap repair, attention should be paid to the following aspects. It is best to use an ultrasound Doppler flow detector to check the perforating vessel for mutations before surgery and the surface projection of the penetration point. The flap pedicle should contain sufficient deep fascia to avoid damaging the pedicle blood vessel. Oppression of the pedicle rotation point is prohibited. Sufficient space should be reserved under the covered skin for film drainage. The cutaneous nerve should be exactly anastomosed with the broken end of the sural nerve to restore the partial sensory function of the flap.

### Authors’ Contributions:

**ZH & ST:** Designed this study and prepared this manuscript, and are responsible and accountable for the accuracy or integrity of the work.

**CH:** Collected and analyzed clinical data.

**YJ:** Significantly revised this manuscript.
